# What is the “modified” CTAB protocol? Characterizing modifications to the CTAB DNA extraction protocol

**DOI:** 10.1002/aps3.11517

**Published:** 2023-06-02

**Authors:** John J. Schenk, L. Ellie Becklund, S. James Carey, Paige P. Fabre

**Affiliations:** ^1^ Department of Environmental and Plant Biology Ohio University Athens Ohio 45701–2979 USA

**Keywords:** cell lysis, cetyltrimethylammonium bromide (CTAB), DNA extraction, high‐throughput sequencing, molecular biology, molecular systematics, plant genetics

## Abstract

Cetyltrimethylammonium bromide (CTAB)–based methods are widely used to isolate DNA from plant tissues, but the unique chemical composition of secondary metabolites among plant species has necessitated optimization. Research articles often cite a “modified” CTAB protocol without explicitly stating how the protocol had been altered, creating non‐reproducible studies. Furthermore, the various modifications that have been applied to the CTAB protocol have not been rigorously reviewed and doing so could reveal optimization strategies across study systems. We surveyed the literature for modified CTAB protocols used for the isolation of plant DNA. We found that every stage of the CTAB protocol has been modified, and we summarized those modifications to provide recommendations for extraction optimization. Future genomic studies will rely on optimized CTAB protocols. Our review of the modifications that have been used, as well as the protocols we provide here, could better standardize DNA extractions, allowing for repeatable and transparent studies.

It was immediately clear at the beginning of the molecular DNA revolution that contemporary protocols for plant DNA extractions were inadequately slow, expensive, and low‐throughput, and that alternative methods were needed (Doyle and Doyle, 1990). Extractions that included the detergent cetyltrimethylammonium bromide (CTAB) seemingly resolved those issues, allowing for fast, inexpensive, and relatively high‐throughput DNA extractions that required only small amounts of input tissue (Doyle and Doyle, 1987). Although CTAB had been used to extract DNA from plants for over three decades prior (Dutta et al., 1953; Murray and Thompson, 1980; Saghai‐Maroof et al., 1984), its use was popularized by Jeff and Jane Doyle (Doyle and Doyle, 1987), who demonstrated its efficacy while outlining how it could be individually optimized for different species. Despite the utility of CTAB‐based extraction methods, extractions from some organisms still failed to produce pure, high‐molecular‐weight DNA (Varma et al., 2007).

Suboptimal DNA extractions are caused by the same anatomical and chemical properties that allow plants to survive in their environments. Rigid cell walls, for example, provide structural support but trap DNA within cells, and their polysaccharides can inhibit downstream molecular approaches, such as PCR amplification, ligation, and enzymatic reactions (Do and Adams, 1991; Pandey et al., 1996; Varma et al., 2007). In addition, the secondary metabolites that protect plants from environmental stresses (Pratyusha, 2022) and, in particular, herbivory are also reactive when tissues are macerated during DNA extractions. Numerous secondary metabolites are present within plant cells, including polysaccharides, phenolic compounds, anthocyanins, alkaloids, saponins, essential oils, and resins, but polysaccharides and phenolic compounds are particularly difficult to remove during the extraction process (Do and Adams, 1991; Wilson, 1997) because they coprecipitate with DNA (Scott and Playford, 1996; Sharma et al., 2002). The optimization of DNA extractions since Doyle and Doyle (1987) has centered on isolating DNA in the face of the substantial obstacles caused by the coprecipitation of polysaccharides and phenolic compounds.

Over 8000 phenolic compounds have been described in plants, including simple phenols, phenolic acids, and polyphenols, among others (Pratyusha, 2022). When cells are disrupted, phenolic compounds are released from the vacuoles and freely undergo oxidative reactions (Pratyusha, 2022), which can either be catalyzed by oxidizing enzymes or occur non‐enzymatically (Loomis and Battaile, 1966). Lysates become discolored as they oxidize, even turning black in some reactions. During oxidation, phenolic compounds covalently bond with DNA, and these bonds cannot be broken with traditional extraction protocols (Loomis and Battaile, 1966; John, 1992; Varma et al., 2007; Abubakar et al., 2018). Consequently, the DNA–phenol complexes formed during oxidation are discarded during the cleaning process due to their high molecular weight (Japelaghi et al., 2011), ultimately yielding little to no DNA.

Polysaccharide and DNA coprecipitation creates two major issues during DNA extractions. First, the reaction becomes viscous with the addition of alcohol, resulting in incorrect pipetted volumes. Second, and more importantly, polysaccharides inhibit downstream molecular reactions (Shioda and Murakami‐Murofushi, 1987; Pandey et al., 1996; Varma et al., 2007). Not all polysaccharides inhibit downstream approaches, however. Neutral polysaccharides are benign in restriction digestions, but acidic polysaccharides, such as carrageenan, gum ghatti, and others, inhibit restriction digests (Do and Adams, 1991).

Preventing polysaccharide and phenolic compound coprecipitation remains the greatest challenge during DNA extractions. DNA extractions received considerable attention near the end of the 20th century, when coprecipitates inhibited commonly used methods at the time, such as gene mapping, Southern blots, genetic fingerprinting, and Sanger sequencing for phylogenetic and population studies. Low yield and highly contaminated DNA extractions, however, are just as problematic today (as evidenced by other articles in this special issue; e.g., Jones et al., 2023; Xie et al., 2023) and will continue to play a prominent role in the future. Coprecipitates, such as polysaccharides and phenolic compounds, inhibit PCR amplification, ligation, and restriction digestion (Do and Adams, 1991; Fang et al., 1992; Pandey et al., 1996) and continue to be problematic for molecular studies. Commonly used genomic approaches, such as genomic library construction for high‐throughput sequencing, require ligation and PCR amplification that can be inhibited by coprecipitates. Gene quantification techniques, such as quantitative PCR, are also impeded by these contaminants (Demeke et al., 2009). Restriction site–associated DNA sequencing (RADseq), a commonly applied reduced representation genomic approach (Baird et al., 2008; Davey and Blaxter, 2010), requires restriction digestion. In RADseq experiments, impurities in the DNA extractions could inhibit enzymatic digestion, resulting in missing loci that could limit inferences of population structure and phylogenetic relationships (Leaché et al., 2015). Next‐generation sequencing approaches, such as Illumina's (San Diego, California, USA) sequencing by synthesis, utilize polymerases (Chen, 2014), which are inhibited by polysaccharides. The coprecipitation of phenolic compounds and polysaccharides might be a factor causing poor high‐throughput sequencing of some species or samples (e.g., Becklund and Ayers, 2022; Cohen and Schenk, 2022; Donoghue et al., 2022), but this hypothesis is yet to be tested. As biologists move toward long‐read genomic sequencing, our capacity to sequence kilobases of nucleotides will be limited by our ability to extract unfragmented and high‐quality DNA (Cerda et al., 2023; Kang et al., 2023; Xie et al., 2023). Optimizing the CTAB protocol to remove secondary metabolites is, therefore, imperative for the future of genomic studies.

The CTAB protocols popularized by Doyle and Doyle (1987, 1990) are currently the most commonly applied DNA extraction protocols, cited over 22,000 times (Google Scholar search on 11 August 2022; Doyle and Doyle, 2019). The authors recognized that plants vary greatly in their chemical compositions and obtaining pure, high‐molecular‐weight DNA is difficult in some species. Consequently, the authors included numerous alternative approaches to successfully extract DNA; we estimate they proposed 24 different ways, not including the varying of incubation times. Although the strength of the Doyle and Doyle (1987) protocol is its versatility, problems arise when research articles cite the paper as their approach but do not explicitly state the conditions under which they extracted DNA (even the lead author is guilty of making this error; Schenk and Hufford, 2011), or the authors state that a “modified” CTAB protocol was performed without detailing the ways in which it was modified. Such scenarios have created a critical problem because those studies are neither transparent nor reproducible, neglecting the fundamental tenets of research articles. Furthermore, the numerous modifications of the CTAB protocol have not been rigorously reviewed. Performing such a review will assist researchers with optimizing their extractions while simultaneously illuminating the conditions of the protocols that are poorly understood.

In our review, we detail the ways in which the CTAB protocol has been modified for plant DNA extractions over the years. To meet this objective, we surveyed a subset of the literature for studies that focused on modifying the CTAB protocol and reported how extraction protocols have varied across those studies. We conclude by providing several protocols (Appendices [Link], [Link]; see Supporting Information with this article) and recommendations for researchers to ensure that future studies are transparent and reproducible.

## DISCUSSION

### The modified CTAB protocol

**Figure 1:**
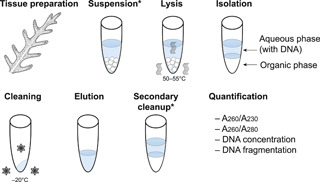
Eight steps of the CTAB extraction protocols, all of which have been modified by researchers across studies. The majority of studies excluded the suspension and secondary cleanup steps, which are denoted with asterisks. Areas in tubes shaded blue denote where the DNA is located and those shaded light gray represent the organic phase during the cleaning steps. The lysis step included the application of heat to facilitate cell and tissue lysis. The cleaning step often, but not always, involved precipitation of the DNA at freezing temperatures.

Through our literature review, we have determined that citing a “modified” CTAB extraction is almost meaningless given the extensive ways the protocol has been modified across studies. In addition to chemical additives to reduce polysaccharide and phenolic compound coprecipitation, the individual steps of the CTAB protocol also have been modified in many ways. Below, we review how researchers have modified the protocol and discuss the importance of those modifications. We recognize eight protocol steps that are applied during CTAB extractions (Figure 1): tissue preparation, suspension, lysis, isolation, cleaning, elution, secondary cleanup, and quantification. Most protocols, however, did not include all eight steps, and often the suspension and secondary cleanup were omitted.

#### Tissue preparation

Both tissue selection and method of storage are vital to the success of DNA extractions. Freshly collected tissues that are frozen in liquid nitrogen tend to perform best (Doyle and Doyle, 1990), but extractions from tissues desiccated in silica gel also produce high‐molecular‐weight and pure DNA (Chase and Hills, 1991). A high‐salt CTAB solution has also been used as an alternative method to store leaf samples that can later be extracted (Rogstad, 1992; Štorchová et al., 2000), as has the use of ethanol storage (Johnson et al., 2023). The best tissues to use are young leaves, as greater yields and purity of DNA have been obtained from them compared to mature leaves, roots, fruits, and stems (Jobes et al., 1995; Japelaghi et al., 2011; Abubakar et al., 2018), although Rogers and Bendich (1985) determined that embryos produced the highest yields. Leaves that have experienced stress, such as herbivory, will likely have mounted a chemical defense that includes secondary metabolites (Usha Rani and Jyothsna, 2010) and should therefore be avoided, especially in groups with high concentrations of phenolic compounds.

Tissues from herbarium specimens have been successfully used in CTAB extractions (Cota‐Sánchez et al., 2006; Staats et al., 2011), including tissues as old as 204 years (Brewer et al., 2019). Extractions based on herbarium tissues are inferior to fresh or silica gel–preserved tissues because of DNA degradation and lower DNA yields (Ribeiro and Lovato, 2007; Staats et al., 2011). Carey et al. (2023) determined that herbarium tissues resulted in DNA extractions with significantly lower yields, more secondary contaminants, and shorter fragment lengths on average compared to silica gel–preserved tissues.

The amount of starting tissue used for DNA extractions varied substantially across protocols. For dried tissue, protocols used anywhere between 0.4–6000 mg (Syamkumar et al., 2003; Listyanto et al., 2021). Most protocols added 1000 mg of leaf tissue, which is similar to Doyle and Doyle (1987), who used 500–1500 mg. The ratio of tissue weight to buffer volume is likely an important, but overlooked, determinant of extraction success, which is discussed in greater detail below.

#### Suspension

An initial suspension buffer, which has sometimes been referred to as an extraction buffer (Li et al., 2001; Esfandani‐Bozchaloyi et al., 2019), was infrequently applied as a pretreatment or grinding buffer. Some protocols called for CTAB in the suspension buffer (Khanuja et al., 1999; Sharma et al., 2002), albeit at a higher concentration (10–20%) than what has typically been applied in a lysis buffer. The suspension buffers were often similar to the lysis buffers, and were just as variable in their contents, containing sorbitol, sucrose, Triton X‐100, polyvinylpyrrolidone (PVP), CTAB, Tris‐HCl, sodium bisulfate, EDTA, and β‐mercaptoethanol (Khanuja et al., 1999; Štorchová et al., 2000; Li et al., 2001; Esfandani‐Bozchaloyi et al., 2019). We also observed protocols that ground the tissues in pure β‐mercaptoethanol (Kim et al., 1997) or PVP (Sahu et al., 2012). The suspension step, when applied, often involved mechanical maceration followed by a short incubation (e.g., 15 min) at room temperature (Sharma et al., 2002), or no incubation, followed by centrifugation and transferral of the supernatant. The protocol of Esfandani‐Bozchaloyi et al. (2019) was quite different in applying a heated incubation during the suspension step and not during the lysis step.

#### Lysis

The lysis step typically involved maceration of the tissues in a lysis buffer and then a heated incubation. We refer to a buffer as a lysis buffer only if it is incubated with heat. Most protocols macerated tissues in lysis buffer immediately after tissue preparation instead of using a suspension buffer. The main function of the lysis step is to disintegrate tissues, cells, and organelles to release their contents, and in particular, the DNA, from the protoplast, nucleus, and organelles. The lysis step, therefore, begins with maceration, which was usually done mechanically but can also be done enzymatically (Manen et al., 2005). Mechanical maceration often involved either bead mills or grinding with a mortar and pestle (Hale et al., 2020), and some approaches called for the addition of sterilized sand to facilitate grinding (Scott and Playford, 1996). Partially ground or dissolved tissues inhibit the extraction of high‐molecular‐weight DNA (Csaikl et al., 1998). Tissues were either ground frozen using liquid nitrogen, or desiccated tissues were ground dry and/or with a suspension or lysis buffer. Studies have concluded that mechanical maceration is superior to chemical maceration (Manen et al., 2005; Aboul‐Maaty and Oraby, 2019), and that grinding with liquid nitrogen to keep fresh tissues frozen resulted in better quality DNA, likely because of reduced reaction time of the phenolic compounds (Lodhi et al., 1994) or nuclease activity (Kasem et al., 2008). For the mechanical separation of tissues, bead mills produced higher‐molecular‐weight DNA than grinding tissues with a mortar and pestle (Drábková et al., 2002).

Lysis buffers included Tris, NaCl, EDTA, CTAB, and β‐mercaptoethanol, as well as many of the additives listed below. EDTA chelates Mg^2+^, which effectively inactivates nucleases, an important step in preventing DNA degradation during the extraction process (Rogers and Bendich, 1985; Puchooa, 2004). Protocols used different concentrations of CTAB. The Doyle and Doyle (1987) protocol called for a 2% CTAB concentration, which is the most common percentage, but modifications ranged between 1–4% (Saghai‐Maroof et al., 1984; Syamkumar et al., 2003). Using higher concentrations of CTAB reduces polysaccharides (Syamkumar et al., 2003), but CTAB is also a PCR inhibitor and can produce artificially high DNA concentration estimates when spectrophotometers are used (Demeke and Jenkins, 2010), necessitating the thorough removal of CTAB from the extractant during the cleaning step.

The concentration of NaCl varied in lysis buffers. Using high concentrations of NaCl in the lysis buffer reduces polysaccharide coprecipitates in the extractant (Syamkumar et al., 2003; Abubakar et al., 2018), but as with high concentrations of CTAB, care must be taken to thoroughly clean the extraction as carryover can inhibit PCR amplification (Demeke and Jenkins, 2010). The Doyle and Doyle (1987) protocol called for 1.4 M NaCl, which is the most common concentration, but protocols ranged between 0.7–4 M (Saghai‐Maroof et al., 1984; Tel‐Zur et al., 1999).

The volume of lysis buffer applied across protocols ranged from 400–16,000 μL (Puchooa, 2004; Larridon et al., 2020), but on average most protocols used ≤2000 μL. To our knowledge, an experiment has not been published that determines the optimal ratio of the lysis buffer volume to tissue weight, evaluated by the purity and concentration of DNA during CTAB extractions. Murray and Thompson (1980) recommended 1 mL of buffer per 30–100 mg of dry weight powder. Puchooa (2004) did not detail their experimental design or what criterion was used, but recommended a 4:1 v/w ratio. We can assume that increased tissue amounts should require greater lysis buffer volume, but the ratio needs to be experimentally determined with explicit criteria based on DNA yield and purity across representative groups. In addition, greater buffer to tissue ratios might be necessary in plants with high concentrations of secondary metabolites, such as those that produce latex (Michiels et al., 2003). To extract under greater buffer to tissue ratios, some protocols advise splitting the lysate into two 1.5‐mL microcentrifuge tubes and then moving to the isolation step (Scott and Playford, 1996), but a smaller amount of starting tissue could alternatively be used to obtain a high buffer to tissue ratio. It is noteworthy that the Doyle and Doyle (1987) protocol applied 8000 μL of a lysis buffer in their reaction. The present‐day use of smaller volumes likely reflects the common usage of microcentrifugation, which allows only 1.5/2.0‐mL microcentrifuge tubes, as many laboratories simply do not have large‐volume centrifuges.

During the lysis step, the lysate is incubated above room temperature to facilitate tissue degradation and cell lysis. Incubation temperature and duration varied greatly among studies. Temperatures ranged between 55–70°C (Scott and Playford, 1996; Arruda et al., 2017), while Doyle and Doyle (1987) applied 60°C. The duration varied between 1–86,400 min (Rogers and Bendich, 1985; Larridon et al., 2020), which differed greatly from Doyle and Doyle's (1987) recommendation of 30–60 min. On average, studies used a temperature of around 60°C and incubated for 60 min. For shorter incubation times (≤30 min), preheating the lysis buffer is necessary (Doyle and Doyle, 1987). Carey et al. (2023) conducted experiments that explored a range of temperatures (50–65°C) and incubation times (1–24 h) and determined that on average cooler temperatures (~50–55°C) and shorter incubation times (1 h) resulted in slightly higher DNA yields, less coprecipitation, and longer DNA fragments, although not all differences in temperature and duration were significant.

Mixing during incubation varied among protocols. Abubakar et al. (2018), for example, shook the tubes every 10 min during incubation, whereas Japelaghi et al. (2011) shook and vortexed every 5 min. Vortexing has been shown to provide some benefits but risks shearing the DNA (Sharma et al., 2000). Li et al. (2001) experimented with vortexing for zero, 20, 40, and 60 s after the lysis buffer was added and determined that 60 s produced the highest yield and that all three vortexing times resulted in higher yields than simply inverting, but the authors did not quantify the extent of DNA fragmentation in their experimental results.

#### Isolation

During the isolation step, DNA is physically separated from all other cellular components that are produced in the lysate during the lysis step. Isolation is achieved in different ways. Recent studies applied a pre‐isolation step where the lysate was combined with a buffer and then filtered to remove large cellular debris (Jones et al., 2023; Xie et al., 2023), but the most common method was with 24:1 v/v chloroform–isoamyl alcohol (CIA). The volume of CIA should match that of the lysis buffer. Earlier CTAB protocols often included phenol, but it might not be necessary for plant tissues, and protocols have excluded it because of health risks (Varma et al., 2007). When the lysate is combined with CIA, the mixture separates into two phases, an upper aqueous and lower organic phase (Figure 1). The solubility of the chemicals in the lysate determines the phase in which they suspend. The lower organic phase includes proteins, lipids, and cellular debris, whereas the upper aqueous phase contains DNA, RNA, and polysaccharides; phenolic compounds are found in both phases. Some protocols (Japelaghi et al., 2011; Hale et al., 2020) call for briefly vortexing to mix the lysate with the CIA, but excessive vortexing should be avoided to prevent the DNA from shearing.

Centrifugation of the CIA and lysate mix facilitates the separation of the aqueous and organic phases and ranged between 8000–14,000 rpm for 0.5–30 min (Rogers and Bendich, 1985; Pirttilä et al., 2001; Cota‐Sánchez et al., 2006; Japelaghi et al., 2011). After centrifugation, the aqueous phase that includes the DNA is pipetted off and transferred into a new tube. One of the most critical steps of the extraction is pipetting the aqueous phase without disturbing the phase transition zone, as accidently doing so will greatly increase carryover.

The isolation step was often repeated when the aqueous phase was not clear (Porebski et al., 1997; Aboul‐Maaty and Oraby, 2019). A second, high‐concentration CTAB lysis buffer that ranged between 5× and 10× was included after isolation in some protocols (Murray and Thompson, 1980; Csaikl et al., 1998; Moreira and Oliveira, 2011). Sharma et al. (2002) recommended that colored pellets should be taken through the isolation and cleanup steps for up to three iterations. For extractions resulting in high concentrations of protein carryover, such as extractions from seeds, at least one extra CIA isolation should be used to remove proteins.

#### Cleaning

The cleaning step could involve the use of commercially available DNA cleaning kits, but more often protocols used less expensive alcohols and sometimes salts. If alcohol and high‐concentration salts are used, the first step is to precipitate the DNA. Protocols either first precipitated the aqueous phase with isopropanol (chilled to –20°C or at room temperature), sometimes with the addition of salts (Aboul‐Maaty and Oraby, 2019), or –20°C EtOH was directly added to the aqueous phase in a separate tube. The earlier protocols that called for the use of a glass rod to remove the DNA after precipitation (e.g., Dutta et al., 1953; Bult et al., 1992) are no longer applied. In our protocols (Appendices S1–S4), the aqueous phase from the isolation stage was combined with 95% EtOH and precipitated overnight at –20°C. Alternative approaches included adding isopropanol and incubating at 25°C overnight, which resulted in lower yields but purer DNA (Michiels et al., 2003), or incubating at –20°C for 10–120 min (Štorchová et al., 2000; Abubakar et al., 2018). Other protocols advocated for the addition of salts with isopropanol to better precipitate the DNA. Tel‐Zur et al. (1999), for example, added 2/3 volume of sodium acetate and isopropanol and then immediately centrifuged, while Porebski et al. (1997) used 5 M NaCl with 95% EtOH chilled to –20°C, which precipitated at –20°C for 10 min to overnight. If high concentrations of salts are added, additional washes are necessary to prevent carryover (Pirttilä et al., 2001). After precipitation, the samples were centrifuged to create a pellet and the supernatant was removed (Figure 1). Some protocols recommended cold centrifugation (~4°C) to maintain the DNA precipitate during centrifugation (Drábková et al., 2002), but others presumably centrifuged at room temperature.

Extractions from herbarium specimens were precipitated for longer time periods, for example, up to two weeks at –20°C in 70% isopropanol (Staats et al., 2011). Hale et al. (2020) recommended precipitating DNA extracted from herbarium material in isopropanol at –20°C for 48 h to two weeks. We have not identified studies that experimentally determined the optimal alcohol, salt concentration, duration, or temperature during DNA precipitation, but experimentally optimizing this critical step could generate higher‐molecular‐weight and purer DNA extractions.

Most protocols incorporated two wash steps in 70–80% EtOH after the DNA was precipitated (Maguire et al., 1994; Štorchová et al., 2000), which is critical to remove the CTAB that is soluble in ~80% EtOH (Rogers and Bendich, 1985), as well as other salts. Washing in EtOH is preferred over isopropanol because solutes, such as sucrose and NaCl, can coprecipitate with isopropanol (Bult et al., 1992). Experimental studies have not been published to determine the best concentration of alcohol, but lower concentrations are likely important in high‐concentration‐salt protocols to reduce carryover. In some studies, the first wash was a higher concentration of alcohol than the second, and sometimes included additional salts to facilitate precipitation (Porebski et al., 1997). Xin and Chen (2012), for example, used Tris‐EDTA (TE) buffer in EtOH.

The final cleanup stage removes all traces of EtOH and/or isopropanol by removing the supernatant with a pipette and then either leaving the tubes open to allow the alcohol to evaporate at room temperature, or briefly applying low heat, sometimes under a vacuum. Removing all traces of alcohol is important as it inhibits PCR amplification (Demeke and Jenkins, 2010). Centrifuging with high force, for too long (Murray and Thompson, 1980), or overdrying the DNA pellet will result in damaged or low‐molecular‐weight DNA and prohibit the DNA pellet from dissolving in the elution buffer (see below).

#### Elution

The DNA pellet needs to be resuspended once it is dried. Protocols generally called for either the use of H_2_O (Moreira and Oliveira, 2011) or 1× TE, the latter of which is preferred to buffer the DNA. The elution volume ranged from 10–800 μL (Demeke and Adams, 1992; Štorchová et al., 2000), but 50–100 μL was most common. Some protocols heat the pellet in the elution buffer at 50–65°C for 5–120 min to resuspend the DNA (Rogers and Bendich, 1985; Aboul‐Maaty and Oraby, 2019), although lower temperatures are more prudent to prevent DNA fragmentation.

#### Secondary cleanup

Some protocols advocated for a post‐extraction secondary cleanup. Secondary cleanup is often applied with commercially available DNA cleaning kits (e.g., Demeke et al., 2009); however, some studies advocated for the use of chemical cleanups, such as cleaning with phenol and chloroform (Tel‐Zur et al., 1999; Michiels et al., 2003), magnetic beads (Larridon et al., 2020), or gel purification (Bult et al., 1992). Historically, ultracentrifugation with a CsCl gradient was employed for secondary cleanups (Doyle and Doyle, 1990; Wilkie et al., 1993), but that approach is no longer applied. If an extraction produced pure, high‐molecular‐weight DNA, a secondary cleanup, which is not mandatory, will only reduce DNA concentrations while wasting time and money.

#### Quantification

Four methods were commonly applied to quantify DNA: spectrophotometers, fluorometers, gel electrophoresis, and visualization of extractant color. Spectrophotometry, such as that applied with a NanoDrop (Thermo Fisher Scientific, Waltham, Massachusetts, USA), was the most common approach, even though DNA concentration estimation is problematic (Drábková et al., 2002). Despite the issues with quantifying DNA concentrations, NanoDrop can quantify coprecipitates in the extractant, including the ratio between the A_260_/A_280_ spectra, which measures the ratio of DNA to proteins, and the ratio between the A_260_/A_230_ spectra, which measures the ratio of DNA to contamination from phenolic compounds and polysaccharides (Varma et al., 2007). Carryover CTAB in the final extractant could increase the A_260_ nm absorbance readings, which will aberrantly inflate the estimated concentration of DNA (Doyle and Doyle, 1990; Wilkie et al., 1993). Consequently, a fluorometer that directly reads the concentration of DNA, such as the Qubit (Thermo Fisher Scientific) system, is preferred. Gel electrophoresis was often incorporated to determine the distribution of fragment lengths in the extractant, but interpreting concentrations from the banding pattern when they form continuous smears is challenging. A fragment analyzer provides a more rigorous approach to quantify the concentrations of fragment lengths compared to gel electrophoresis (Carey et al., 2023), as it quantifies concentrations and fragment lengths; however, it significantly increases the cost per sample compared to other quantification methods. The color of the extractant also indicates DNA quality (Varma et al., 2007); for example, pigmented extractants indicate phenol‐mediated oxidation (Esfandani‐Bozchaloyi et al., 2019). In our experience, darker extractants were unlikely to work in downstream applications, such as PCR; others have reported similar results (Esfandani‐Bozchaloyi et al., 2019).

### Additives

Chemical additives have been added to the suspension or lysis buffer to reduce polysaccharide coprecipitates and inhibit or remove phenols, and we outline the most commonly used additives below. Less frequently used additives have also been reported, but are not discussed here; these include ascorbic acid, bovine serum albumin, diethyldithiocarbamic acid, dithiothreitol, and sodium azide (see references in Puchooa, 2004; Varma et al., 2007). The four protocols provided in the Supporting Information include a basic CTAB protocol (Appendix S1) and three CTAB protocols that include the additives PVP (Appendix S2), sodium dodecyl sulphate (SDS) (Appendix S3), and sorbitol (Appendix S4). The CTAB, CTAB + PVP, and CTAB + SDS protocols apply six out of the eight steps—excluding the suspension and secondary cleanup steps. The CTAB + sorbitol protocol applies seven of the eight steps—excluding the secondary cleanup, but including a suspension step with a suspension buffer composed of sorbitol, Tris‐Cl, EDTA, and β‐mercaptoethanol.

#### β‐mercaptoethanol

The antioxidant β‐mercaptoethanol is applied in DNA extractions to prevent protein oxidation and denature proteins and was included in the Doyle and Doyle (1987) protocol. β‐mercaptoethanol is most commonly added to the CTAB lysis buffer. The concentrations of β‐mercaptoethanol ranged from 0% (Listyanto et al., 2021) to 5% (Arruda et al., 2017), but most protocols use 0.2–0.5%, which is similar to Doyle and Doyle's (1987) usage of 0.2%. The Fairlie and Pokorny protocol (cited in Larridon et al., 2020) recommended an increase of β‐mercaptoethanol from 0.2% to 0.4% when extracting DNA from herbarium specimens. Silva (2010) experimentally determined that higher concentrations of β‐mercaptoethanol, in their case 1–5%, resulted in cleaner and higher yields of DNA; however, any adverse effects of using high concentrations of β‐mercaptoethanol on downstream reactions are unknown, and further study is needed. β‐mercaptoethanol's toxicity and off‐putting smell has led many laboratories to omit its use in extractions (Listyanto et al., 2021); nevertheless, it does serve an important function in plant DNA extractions by degrading and removing proteins and preventing oxidation (Abubakar et al., 2018).

#### Polyvinylpyrrolidone (PVP)

PVP is an inert, high‐molecular‐weight polymer that forms hydrogen bonds with phenols, inhibiting their reactions and facilitating their removal (Loomis and Battaile, 1966; John, 1992). Compared to other additives, PVP is highly effective at removing phenols and preventing oxidation (Loomis and Battaile, 1966; Rogers and Bendich, 1985; Doyle and Doyle, 1990). Nazhad and Solouki (2008) determined that the addition of PVP increased final DNA concentrations. Compared to sorbitol and SDS additives, Carey et al. (2023) determined that the addition of PVP resulted in the highest yield with less coprecipitates. PVP is used at 1–2.5% in the lysis or suspension buffer (Cullings, 1992; Michiels et al., 2003; Sahu et al., 2012; Arruda et al., 2017). Some studies have used the molecularly similar polyvinylpolypyrrolidone (PVPP) as an alternative, but those extractions produced lower yields than those that used PVP (Lodhi et al., 1994; Porebski et al., 1997). Two forms of PVP were typically used, PVP‐40 (m.w. 40,000; e.g., Doyle and Doyle, 1990) and PVP‐10 (m.w. 10,000; e.g., Puchooa, 2004). Although PVP‐40 is more commonly applied, the use of lower‐molecular‐weight PVP‐10 resulted in higher DNA yield and did not coprecipitate with the DNA (Aljanabi et al., 1999; Puchooa, 2004; Varma et al., 2007). The PVP solution should be made fresh at the beginning of each extraction (John, 1992) and could require heat to dissolve (Kistler, 2012).

#### Proteinase K

The enzyme proteinase K inactivates proteins by breaking peptide bonds. Proteinase K denatures histones and prevents DNase‐ and RNase‐mediated oxidation of DNA or RNA, respectively (Varma et al., 2007). Therefore, although some protocols exclude it, proteinase K plays an important role during the extraction process and should be included in the lysis buffer. Some variation exists in the amount of proteinase K added, which has ranged from 1% of 1 mg/mL to 0.625% of 20 mg/mL in the lysis buffer (Porebski et al., 1997; Demeke et al., 2009).

#### RNase A

RNase breaks down RNA into ribonucleosides, inhibiting the coprecipitation of RNA with DNA during CTAB extractions (Zamboni et al., 2008). Some protocols do not use RNase A (Listyanto et al., 2021), but the presence of RNA could interfere with primer sites during PCR amplification (Jobes et al., 1995; Porebski et al., 1997). The application of RNase A occurred at different steps in the extraction protocol: before the EtOH wash step (Demeke et al., 2009) or after the elution stage (Abubakar et al., 2018). Studies varied in the amount of RNase used—from 0.5% of 34 μg/mL to 1% of 10 mg/mL (Cota‐Sánchez et al., 2006; Demeke et al., 2009)—with the latter concentration being more common than the former. Although RNA is degraded by RNase, the ribonucleosides are not removed from the extractant. Lithium chloride, which completely removes RNA and ribonucleosides (Pirttilä et al., 2001), has been used as an alternative to RNase (Ribeiro and Lovato, 2007).

#### Salts

High concentrations of salts reduce the concentration of polysaccharides in the extractant (Fang et al., 1992; Varma et al., 2007; Abubakar et al., 2018), increasing the purity of the DNA in the extractant (Scobeyeva et al., 2018). Fang et al. (1992) determined that 1 M NaCl was effective at removing polysaccharides in some groups, but tissues that contained high polysaccharide concentrations required 1.5 to 2.0 M. One study increased concentrations to 6 M NaCl (Aljanabi and Martinez, 1997). High salt concentrations, however, are difficult to remove from the final extractant, and they can inhibit downstream applications, such as PCR amplification (Demeke and Jenkins, 2010). Most protocols called for sodium chloride, but potassium acetate, sodium acetate, and sodium metabisulfite have also been included (Aljanabi et al., 1999; Pirttilä et al., 2001; Scobeyeva et al., 2018).

#### Sarkosyl

Sarkosyl is an amphiphilic detergent. In groups with high polysaccharide concentrations, such as Cactaceae, the use of sarkosyl might be vital to remove polysaccharides from the extractant (Tel‐Zur et al., 1999; Sharma et al., 2002). For polysaccharide removal, a 5% sarkosyl solution has been added to the lysis buffer as 10–20% of the total lysis buffer volume (Scott and Playford, 1996; Li et al., 2001).

#### Sodium dodecyl sulphate (SDS)

SDS is another amphiphilic detergent that breaks down cells and denatures proteins (Kasem et al., 2008). SDS forms complexes with proteins and polysaccharides (Ramachandran et al., 2022), allowing the high‐molecular‐weight complexes to be easily removed. Ramachandran et al. (2022) determined that an addition of 5% SDS to a CTAB buffer outperformed other CTAB protocols that did not include it, but the extractants were not as pure because of carryover. Carey et al. (2023) also determined that the addition of SDS resulted in high carryover, but the DNA yield was not as high as a protocol that included only CTAB or CTAB + PVP. SDS at high concentrations can potentially shear the DNA (Jobes et al., 1995), but the extent of fragmentation has not been experimentally quantified. Most studies that include SDS do so in the lysis or suspension buffer at lower concentrations. Kotchoni et al. (2011), for example, recommended an addition of 1% SDS in the buffer from a 10% w/v stock solution when extracting DNA from *Arabidopsis* Heynh.

#### Sorbitol

Sorbitol is a sugar alcohol that acts as a reducing agent and, at high concentrations, inhibits oxidation from polyphenols (Stein, 1993; Kasem et al., 2008). Sorbitol was first used by Scott and Playford (1996), and its success at reducing oxidation during the extraction has led to its application in other labs (Štorchová et al., 2000). Approximately 0.35 M sorbitol is typically applied in studies (Drábková et al., 2002), usually in a suspension buffer (Štorchová et al., 2000). Li et al. (2001) determined that the addition of sorbitol followed by vortexing increased DNA yield. A study by Carey et al. (2023) determined that sorbitol had higher contamination and lower yield than extractions with CTAB only or CTAB + PVP, but it had the largest average fragment size, which might make it appealing for long‐read sequencing.

### Alternatives to CTAB

CTAB is not the only method for the isolation of DNA from plants. Numerous commercial kits promise rapid extractions that result in high‐purity DNA. However, in all but one study that we came across (albeit with a modified protocol, Drábková et al., 2002), CTAB outperformed commercially available DNA extraction kits in generating greater yields, and in most cases, higher‐quality DNA. CTAB performed better than DNeasy Plant Mini Kit (Qiagen, Valencia, California, USA), DNP Kit (CinnaGen, Tehran, Iran), NucleoSpin Plant II (Machery‐Nagel, Düren, Germany), GeNei (Genei Laboratories, Peenya, India), and Wizard Resin (Promega, Madison, Wisconsin, USA) (Michiels et al., 2003; Demeke et al., 2009; Sahu et al., 2012; Abubakar et al., 2018; Esfandani‐Bozchaloyi et al., 2019). The advantage of the CTAB protocol over commercial kits is that it produces higher DNA yield and quality and each extraction is less expensive (Hale et al., 2020).

Non‐commercial protocols that do not include CTAB have successfully extracted high‐quality, high‐molecular‐weight DNA, including those based on PVP (Guillemaut and Maréchal‐Drouard, 1992; Jobes et al., 1995; Ribeiro and Lovato, 2007), sorbitol (Scott and Playford, 1996; Ribeiro and Lovato, 2007), and SDS (Dellaporta et al., 1983; Jobes et al., 1995; Sharma et al., 2000; Hosaka, 2004; Ribeiro and Lovato, 2007; El Maaiden et al., 2019). In a study that evaluated the above methods in the tropical genus *Dalbergia* L.f. (Fabaceae), Ribeiro and Lovato (2007) determined the PVP‐only protocol of Jobes et al. (1995) and a CTAB protocol outperformed the others, whereas Csaikl et al. (1998) determined that two different CTAB protocols outperformed the SDS‐only protocols. An alternative method based on diethyldithiocarbamic acid was shown to be effective in the phenolic‐rich *Theobroma cacao* L. (Couch and Fritz, 1990), but the study did not include a comparison to CTAB. Enzymatic reactions applying a variety of enzymes that break down tissues and cells (e.g., cellulase, pectinase, glycosidase) are another approach that could also facilitate DNA extraction automation (Manen et al., 2005); however, enzymatic approaches require significant preparation and were not commonly applied. In ancient DNA samples, the use of *N*‐phenacylthiazolium bromide resulted in high‐quality extractions in Cucurbitaceae fruits (Kistler, 2012) and other degraded material (Bernardo et al., 2005).

### Recommendations and future directions

Properly cited DNA extraction protocols that explicitly state how they were modified are paramount (e.g., Demeke et al., 2009). Rather than stating “we extracted DNA using a modified CTAB protocol (Doyle and Doyle, 1987),” we should provide specific language, for example: “we extracted DNA with the Doyle and Doyle (1987) protocol except we repeated the isolation step twice.” This alternative is more informative and allows for the study to be reproducible, while remaining concise. Protocols with several modifications should be included in the supplemental materials, as we have done here (Appendices [Link], [Link]). As a community, we must do better to accurately report our experimental procedures and require our colleagues do the same during peer review.

Although CTAB remains one of the most common extraction methods, it is still relatively low‐throughput. A recent study attempted to create a high‐throughput CTAB‐based protocol with some success (Xin and Chen, 2012), although it is not commonly applied. The Xin and Chen (2012) approach was notable in incorporating magnetic beads for cleaning an entire 96‐well plate; however, the need for a high‐throughput extraction protocol remains.

Additional research is needed to further explore how to best optimize CTAB DNA extractions. Those studies need to apply explicit criteria to evaluate optimization to address remaining issues, such as the presence of secondary metabolites, DNA yield, and average DNA fragment lengths. In particular, studies are needed to investigate the optimal tissue to buffer ratio, concentration of β‐mercaptoethanol, vortex duration (quantified with average DNA fragment length), precipitation duration, precipitation temperature, concentrations and forms of alcohol and associated salts during precipitation, alcohol concentrations during washing, and secondary metabolite inhibition on library construction and next‐generation sequencing (e.g., sequencing by synthesis). Although a universal extraction protocol still remains unlikely, optimization experiments can improve our ability to achieve better extractions across plant groups.

## AUTHOR CONTRIBUTIONS

The study was envisioned and designed by L.E.B. and J.J.S., and the manuscript was written by J.J.S., L.E.B., S.J.C., and P.P.F. All authors approved the final version of the manuscript.

## Supporting information


**Appendix S1**. Total genomic DNA extraction from plant tissue using CTAB.

**Appendix S2**. Total genomic DNA extraction from plant tissue using CTAB + PVP.

**Appendix S3**. Total genomic DNA extraction from plant tissue using CTAB + SDS.

**Appendix S4**. Total genomic DNA extraction from plant tissue using CTAB + sorbitol.
