# Supplementary material for: What is the “modified” CTAB protocol? Characterizing modifications to the CTAB DNA extraction protocol
Source: Appl Plant Sci. 2023 Jun 2;11(3):e11517. doi: 10.1002/aps3.11517 (PMC10278931; doi:10.1002/aps3.11517)
Supplement: Supplementary file 1 — Appendix S1. Total genomic DNA extraction from plant tissue using CTAB. [file APS3-11-e11517-s004.docx]

**Appendix S1.** Total genomic DNA extraction from plant tissue using CTAB.

Schenk Lab, Version 4

Last Updated 10/16/2021

Composed by K. Marlowe, updated by J. Schenk and E. Becklund

**Materials and Equipment**

Materials:

1. Tris base (CAS: 77-86-1)

2. NaCl (CAS: 7647-14-5)

3. EDTA (CAS: 6381-92-6)

4. CTAB (cetyltrimethylammonium bromide, CAS: 57-09-0)

5. Ultrapure H_2_O (product no. 10977-015; Thermo Fisher Scientific, Waltham, Massachusetts, USA)

6. β-mercaptoethanol (CAS: 60-24-2)

7. EtOH (CAS: 65-17-5)

8. 24:1 chloroform–isoamyl alcohol (CIA)

9. 1X Tris-EDTA (TE) buffer (e.g., product no. BP2473-100; Thermo Fisher Scientific)

10. Proteinase K (CAS: 39450-01-6)

11. 1.5-mL microcentrifuge tubes and rack

12. SYBR Safe DNA Gel Stain (product no. S33102; Thermo Fisher Scientific)

13. Agarose

14. TBE buffer

Equipment:

1. Fisherbrand Bead Mill 24 Homogenizer (Thermo Fisher Scientific)

2. Analog vortex mixer

3. Horizontal centrifuge with refrigeration that reaches 13,000 rpm (e.g., model no. 5430 R; Eppendorf, Hamburg, Germany)

4. Heat block that reaches 50–55°C

5. –20°C freezer

6. Micropipettes and tips

7. 250-mL beaker for waste

8. CentriVap (e.g., CentriVap Micro IR; Labconco, Kansas City, Missouri, USA)

9. Gel electrophoresis (e.g., Owl Easycast B1; Thermo Fisher Scientific)

**Lysis buffer (CTAB)**

Combine

100 mL of 1 M Tris, pH 8.0

280 mL of 5 M NaCl

40 mL of 0.5 M EDTA

20 g of CTAB

Bring up to 1 L with dH_2_O

**To do ahead of time:**

1. Turn on heat block to 50°C.

2. Add appropriate amount of β-mercaptoethanol to CTAB right before using (see p. 5 for calculations).

3. Weigh out 5–10 mg of dry leaf tissue. Place the tissue in a labeled 1.5-mL Bead Mill tube with 2.8-mm ceramic beads that are designated for the Bead Mill (or other bead grinder) in Extraction Step 1.

4. Prepare stocks of 95% and 75% ethanol with ultrapure water chilled to –20°C.

**Lysis and isolation steps:**

1. Place tubes with beads and tissues in the bead mill. Balance your samples. Set speed to S = 3.25 m/s, with other settings at 22°C, C = 01, D = 0:10. Grind dry material for 1 min (T = 1 min).

1. Repeat Extraction Step 1 and grind the tissues for an additional minute.
2. Add 500 μL of CTAB lysis buffer plus β-mercaptoethanol to the dry tissue in each tube in the fume hood. Run samples in the Bead Mill for another minute under the above settings.
3. Remove the samples from the Bead Mill. Vortex refrigerated proteinase K to dissolve any precipitate and then spin for ~1 s in the microcentrifuge. Add 4.0 μL of proteinase K to each sample and briefly pipette mix or vortex and spin to condense liquid at the bottom of tube.
4. Incubate samples for 1 h at 50°C in a heat block.
5. After incubation, transfer digested extract to a newly labeled 1.5-mL tube. Add 500 μL of 24:1 CIA to each tube under the fume hood.
6. Vortex for 10 s and spin at 13,000 rpm for 5 min at room temperature (~21°C).
7.
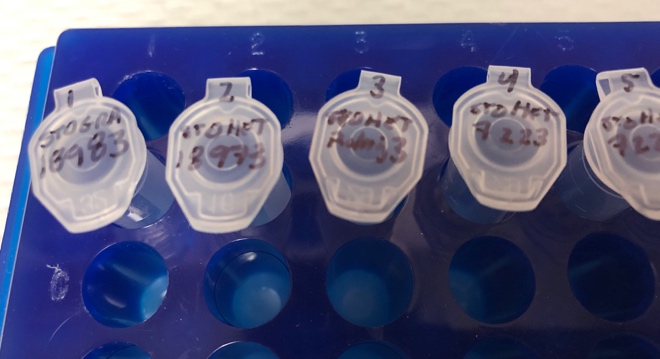
Label a new set of sterile 1.5-mL microcentrifuge tubes (Sample number [on hinge], taxon, and collection or ID number; see image below for an example) and place them into a rack.
8. Remove the tubes from the centrifuge carefully to not disrupt phases. Remove the aqueous phase supernatant with a pipette and transfer the supernatant to the newly labeled tubes from Step 8, being careful not to disrupt the phase transition zone. If you pipette or disrupt the lower organic phases, remix the solution by vortexing and repeat Extraction Step 7. After removing supernatant, discard the old tube into a waste container in fume hood.


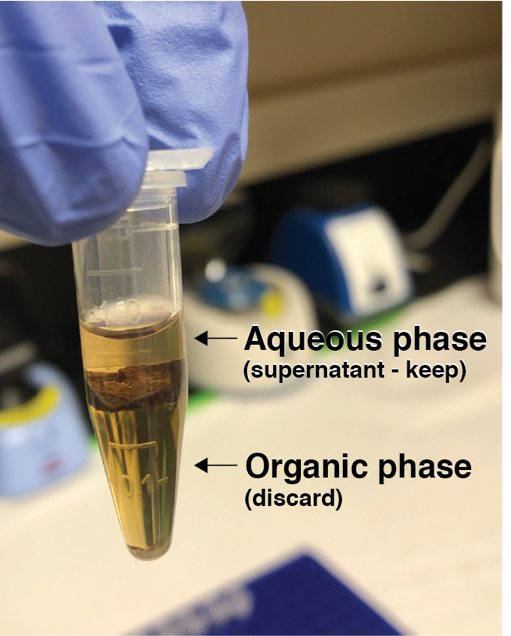


**Cleaning step:**

1. Add 1 mL of 95% ethanol chilled to –20°C to the aqueous phase of each sample. Precipitate DNA overnight at –20°C.

2. Cool down horizontal centrifuge before the next step, then set to 4°C.

3. Spin samples in centrifuge at 4°C for 20 min at 13,000 rpm with the hinge of the tube's lid toward the outside to know where the DNA pellet will concentrate (see images below). After centrifuging, place the tubes in a cold block to keep the pellet frozen.


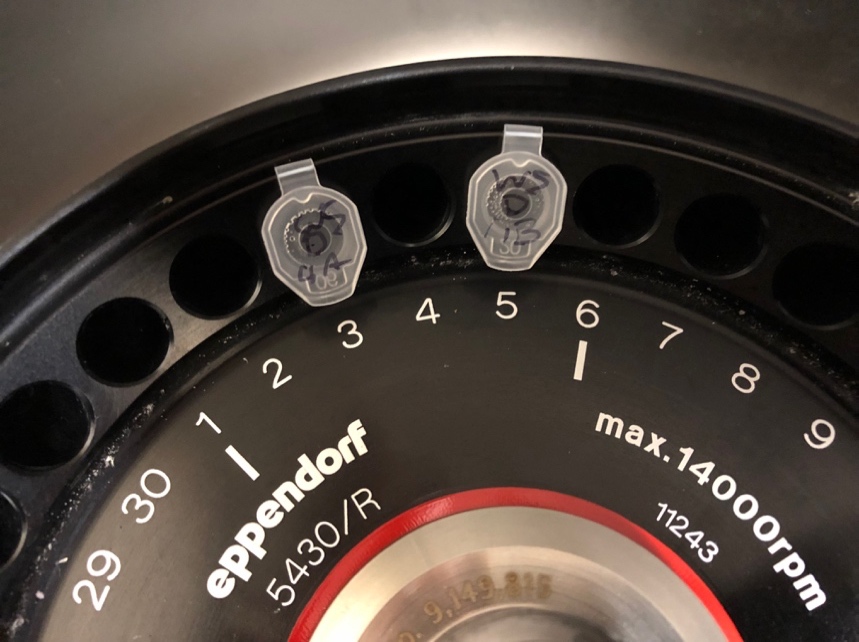

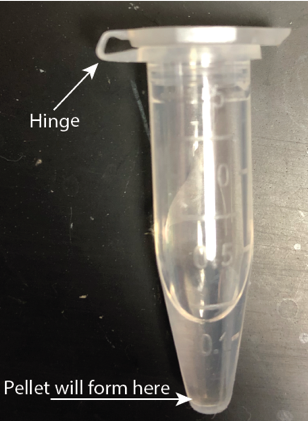


4. Pipette off ethanol into a beaker, being careful not to dislodge the DNA pellet that is located towards the hinge-side of the bottom of the tube (you may or may not be able to see the pellet). Add 500 μL of –20°C 75% ethanol (stored in the –20°C freezer), and spin at 13,000 rpm for 5 min at 4°C. Start heating the CentriVap centrifuge on the standby mode set to 65°C for Step 6 below.

5. Repeat Step 4 for a second wash, then proceed to Step 6.

6. Pipette off as much ethanol as possible, being careful not to disrupt the DNA pellet, then place the tubes with their lids open in the SpeedVac set at 65°C and run it for 3 min or until dry. **Do not overdry the pellets**; they should not turn white, but you should not see or smell ethanol.

**Elution and quantification steps:**

1. Add 50 μL of 1X TE buffer to each sample, pipetting up and down to dislodge and mix the pellet.

2. Resuspend the pellet at 50°C for 30 min if needed to dissolve the pellet.

3. Run a 0.8% agarose test gel infused with SYBR Safe to visualize the extraction.

4. Quantify DNA using Qubit using a high-sensitivity kit. If the concentrations are too large for the machine to read with the high-sensitivity kit (>100 ng/μL), use the broad range kit.

5. Resuspended DNA should be stored in the –20°C freezer for current use or in the –80°C freezer for longer-term storage.

**Adding 0.5% β-mercaptoethanol:**

**Ratio of CTAB : β-merc.** = 100 mL : 500 μL or 100,000 μL : 500 μL (200 : 1)

100,000 μL CTAB + 500 μL β-merc. = 100,500 μL total volume

Calculate the total volume needed:

Number of reactions × 500 μL + 250 μL = Y μL (250 μL is to account for pipetting error)

100,500 = Z

Y

100,000 μL = A μL (this is the amount of CTAB needed)

Z

500 μL = B μL (this is the amount of β-merc. needed)

Z

A + B should equal Y; A/B should equal 200 (the ratio)

Examples:

| 8 Samples | 16 Samples | 24 Samples |
| --- | --- | --- |
| 8(500μL) + 250 μL = 4250 μL  100,500 μL = 23.647 = Z  4250  100,000 μL = 4228.855 μL CTAB  23.647  500 μL = 21.144 μL β-merc.  23.647  Combine  ~4229 μL CTAB and ~21 μL β-merc. | 16(500μL) + 250 μL = 8250 μL  100,500 μL = 12.182 = Z  8250  100,000 μL = 8208.955 μL CTAB  12.182  500 μL = 41.045 μL β-merc.  12.182  Combine  ~8209 μL CTAB and ~41 μL β-merc. | 24(500μL) + 250 μL = 12,250 μL  100,500 μL = 8.204 = Z  12,250  100,000 μL = 12,189.055 μL CTAB  8.204  500 μL = 60.945 μL β-merc.  8.204  Combine  ~12,189 μL CTAB and ~61 μL β-merc. |

**Ratio of CTAB : β-merc., 2–24 samples** (24 is maximum number of samples for Bead Mill)

|  | **Y** | **Z** | **A = CTAB** | **B = BME** | **A+B** | **A = CTAB** | **B = BME** |
| --- | --- | --- | --- | --- | --- | --- | --- |
| **Sample #** | **#** × **500 μL** **+ 250 μL** | **100,500 μL/Y** | **100,000 μL/Z** | **500 μL/Z** | **A+B = Y** | **A Rounded** | **B Rounded** |
| 2 | 1250 | 80.4 | 1243.781095 | 6.218905473 | 1250 | 1244 | 6 |
| 3 | 1750 | 57.42857143 | 1741.293532 | 8.706467662 | 1750 | 1741 | 9 |
| 4 | 2250 | 44.66666667 | 2238.80597 | 11.19402985 | 2250 | 2239 | 11 |
| 5 | 2750 | 36.54545455 | 2736.318408 | 13.68159204 | 2750 | 2736 | 14 |
| 6 | 3250 | 30.92307692 | 3233.830846 | 16.16915423 | 3250 | 3234 | 16 |
| 7 | 3750 | 26.8 | 3731.343284 | 18.65671642 | 3750 | 3731 | 19 |
| **8** | **4250** | **23.64705882** | **4228.855721** | **21.14427861** | **4250** | **4229** | **21** |
| 9 | 4750 | 21.15789474 | 4726.368159 | 23.6318408 | 4750 | 4726 | 24 |
| 10 | 5250 | 19.14285714 | 5223.880597 | 26.11940299 | 5250 | 5224 | 26 |
| 11 | 5750 | 17.47826087 | 5721.393035 | 28.60696517 | 5750 | 5721 | 29 |
| 12 | 6250 | 16.08 | 6218.905473 | 31.09452736 | 6250 | 6219 | 31 |
| 13 | 6750 | 14.88888889 | 6716.41791 | 33.58208955 | 6750 | 6716 | 34 |
| 14 | 7250 | 13.86206897 | 7213.930348 | 36.06965174 | 7250 | 7214 | 36 |
| 15 | 7750 | 12.96774194 | 7711.442786 | 38.55721393 | 7750 | 7711 | 39 |
| **16** | **8250** | **12.18181818** | **8208.955224** | **41.04477612** | **8250** | **8209** | **41** |
| 17 | 8750 | 11.48571429 | 8706.467662 | 43.53233831 | 8750 | 8706 | 44 |
| 18 | 9250 | 10.86486486 | 9203.9801 | 46.0199005 | 9250 | 9204 | 46 |
| 19 | 9750 | 10.30769231 | 9701.492537 | 48.50746269 | 9750 | 9701 | 49 |
| 20 | 10250 | 9.804878049 | 10199.00498 | 50.99502488 | 10250 | 10199 | 51 |
| 21 | 10750 | 9.348837209 | 10696.51741 | 53.48258706 | 10750 | 10697 | 53 |
| 22 | 11250 | 8.933333333 | 11194.02985 | 55.97014925 | 11250 | 11194 | 56 |
| 23 | 11750 | 8.553191489 | 11691.54229 | 58.45771144 | 11750 | 11692 | 58 |
| 24 | 12250 | 8.204081633 | 12189.05473 | 60.94527363 | 12250 | 12189 | 61 |
